# The Pre-Cancerous Stage of Cancer

**Published:** 1882-07

**Authors:** Jonathan Hutchinson


### THE PRE-CANCEROUS STAGE OF CANCER.

The patient who has just left the theatre is the subject of cancer of the tongue in an advanced stage. As I demonstrated to you, the lymphatic glands are already enlarged. It is hopeless to think of an operation, and there is nothing before him but death, preceded and produced by a few months of great and continuous suffering. His case, I am sorry to say, is but an example of what is very common. Not a month passes but a case of cancer of the tongue presents itself in this condition. The cases which come whilst the disease is still restricted to the tongue itself are comparatively few; nor does this remark apply only to the tongue. "Too late! Too late!" is the sentence written but too legibly on three fourths of the cases of external cancer concerning which the operating surgeon is consulted. It is a most lamentable pity that it should be so, and the bitterest reflection of all is, that usually a considerable part of the precious time which has been wasted has been passed under professional observation and illusory treatment. In the present instance, the poor fellow has been three months in a large hospital and a month under private care. I feel free, gentlemen, to speak openly on this matter, because my conscience is clear that I have never failed when opportunity offered, both here and elsewhere, to enforce the doctrine of the local origin of most forms of external or surgical cancer, and the paramount importance of early operation. I have tried every form of phraseology that I could devise, as likely to impress this lesson. Nearly twenty years ago I spoke to your predecessors in this theatre concerning the "successful cultivation of cancer"; telling them how, if they wished their patients to die miserably of this disease, they could easily bring it about. The suggestion was, that all suspicious sores should be considered to be syphilitic, and treated internally by iodide of potassium, and locally by caustics, until the diagnosis became clear. More recently I have often explained and enforced the doctrine of a pre-cancerous stage of cancer, in the hope that, by its aid, a better comprehension of the importance of adequate and early treatment might be obtained. According to this doctrine, in most cases of cancer of the penis, lip, tongue, skin, etc., there is a stage—often a long one—during which a condition of chronic inflammation only is present, and upon this the cancerous process becomes engrafted. I feel quite sure that the fact is so. Phimosis and the consequent balanitis lead to cancer of the penis; the soot-wart becomes cancer of the scrotum; the pipe-sore passes into cancer of the lip; and the syphilitic leucoma of the tongue which has existed in a quiet state for years, at length, in more advanced life, takes on cancerous growth. The frequency with which old syphilitic sores become cancerous is very remarkable; on the tongue, in particular, cancer is almost always preceded by syphilis, and hence one of the commonest causes of error in diagnosis and procrastination in treatment. The surgeon diagnoses syphilis, the patient admits the charge, and iodide of potassium seems to do good; and thus months are allowed to slip by in a state of fool's paradise. The diagnosis, which was right at first, becomes in the end a fatal blunder, for the disease which was its subject has changed its nature. I repeat that it is not possible to exaggerate the clinical and social importance of this doctrine. A general acceptance of the belief that cancer usually has a pre-cancerous stage, and that this stage is the one in which operations ought to be performed, would save many hundreds of lives every year. It would lead to the excision of all portions of epithelial or epidermic structure which have passed into a suspicious condition. Instead of looking on whilst the fire smouldered, and waiting till it blazed up, we should stamp it out on the first suspicion. What is a man the worse if you have cut away a warty sore on his lip, and, when you come to put sections under the microscope, you find no nested cells? If you have removed a painful, hard-based ulcer of the tongue, and with it perhaps an eighth part of the organ; and, when all is done, and the sore healed, a zealous pathological friend demonstrates to you that the ulcer is not cancerous, need your conscience be troubled? You have operated in the pre-cancerous stage, and you have probably effected a permanent cure of what would soon have become an incurable disease. I do not wish to offer any apology for carelessness, but I have not in this matter any fear of it.—JONATHAN HUTCHINSON, in *Brit. Med. Journal.*

